# Dual inhibition of Wnt and Yes‐associated protein signaling retards the growth of triple‐negative breast cancer in both mesenchymal and epithelial states

**DOI:** 10.1002/1878-0261.12167

**Published:** 2018-02-21

**Authors:** Andrew Sulaiman, Sarah McGarry, Li Li, Deyong Jia, Sarah Ooi, Christina Addison, Jim Dimitroulakos, Angel Arnaout, Carolyn Nessim, Zemin Yao, Guang Ji, Haiyan Song, Suresh Gadde, Xuguang Li, Lisheng Wang

**Affiliations:** ^1^ Department of Biochemistry, Microbiology and Immunology Faculty of Medicine University of Ottawa Canada; ^2^ China‐Canada Centre of Research for Digestive Diseases University of Ottawa Canada; ^3^ Institute of Digestive Diseases Longhua Hospital Shanghai University of Traditional Chinese Medicine China; ^4^ Ottawa Institute of Systems Biology University of Ottawa Canada; ^5^ Centre for Cancer Therapeutics Ottawa Hospital Research Institute Canada; ^6^ Centre for Biologics Evaluation, Biologics and Genetic Therapies Directorate Health Canada Sir Frederick G. Banting Research Centre Ottawa Canada; ^7^ Regenerative Medicine Program Ottawa Hospital Research Institute Canada

**Keywords:** cancer stem cell, epithelial, mesenchymal, plasticity, triple‐negative breast cancer, Wnt, YAP

## Abstract

Triple‐negative breast cancer (TNBC), the most refractory subtype of breast cancer to current treatments, accounts disproportionately for the majority of breast cancer‐related deaths. This is largely due to cancer plasticity and the development of cancer stem cells (CSCs). Recently, distinct yet interconvertible mesenchymal‐like and epithelial‐like states have been revealed in breast CSCs. Thus, strategies capable of simultaneously inhibiting bulk and CSC populations in both mesenchymal and epithelial states have yet to be developed. Wnt/β‐catenin and Hippo/YAP pathways are crucial in tumorigenesis, but importantly also possess tumor suppressor functions in certain contexts. One possibility is that TNBC cells in epithelial or mesenchymal state may differently affect Wnt/β‐catenin and Hippo/YAP signaling and CSC phenotypes. In this report, we found that YAP signaling and CD44^high^/CD24^−/low^ CSCs were upregulated while Wnt/β‐catenin signaling and ALDH+ CSCs were downregulated in mesenchymal‐like TNBC cells, and vice versa in their epithelial‐like counterparts. Dual knockdown of YAP and Wnt/β‐catenin, but neither alone, was required for effective suppression of both CD44^high^/CD24^−/low^ and ALDH+ CSC populations in mesenchymal and epithelial TNBC cells. These observations were confirmed with cultured tumor fragments prepared from patients with TNBC after treatment with Wnt inhibitor ICG‐001 and YAP inhibitor simvastatin. In addition, a clinical database showed that decreased gene expression of Wnt and YAP was positively correlated with decreased ALDH and CD44 expression in patients’ samples while increased patient survival. Furthermore, tumor growth of TNBC cells in either epithelial or mesenchymal state was retarded, and both CD44^high^/CD24^−/low^ and ALDH+ CSC subpopulations were diminished in a human xenograft model after dual administration of ICG‐001 and simvastatin. Tumorigenicity was also hampered after secondary transplantation. These data suggest a new therapeutic strategy for TNBC via dual Wnt and YAP inhibition.

**Abbreviations**
ALDH: aldehyde dehydrogenase
CSC: cancer stem cell
DEAB: *N*,*N*‐diethylaminobenzaldehyde
EMT: epithelial‐to‐mesenchymal transition
FDA: Food and Drug Administration
HMG‐CoA: 3‐hydroxy‐3‐methyl‐glutaryl‐coenzyme A
MET: mesenchymal to epithelial transition
PDX: patient‐derived xenograft
PP2A: protein phosphatase 2
shRNA: short hairpin RNA
siRNA: short interfering RNA
TNBC: triple‐negative breast cancer
YAP: Yes‐associated protein

## 1 Introduction

Breast cancer remains a leading cause of death in women worldwide (Siegel *et al*., 2016). Triple‐negative breast cancer (TNBC) accounts for 15–20% of all breast cancer, but is disproportionally associated with the majority of breast cancer‐related deaths (Anders and Carey, 2009; Bauer *et al*., 2007). Chemotherapy is currently the mainstay of systemic medical treatment for TNBC and is associated with severe normal tissue toxicity, rapid drug resistance, cancer stem cell (CSC) enrichment, and disease relapse (Jia *et al*., 2016). Hence, development of effective treatments for TNBC is an important unmet medical need.

Tumor plasticity is thought to drive metastasis and tumor relapse (Beerling *et al*., 2016). E‐cadherin is an epithelial marker and an indicator for epithelial‐to‐mesenchymal transition (EMT) and its reverse process, MET (Liu *et al*., 2014). Epithelial breast CSCs are capable of converting into the mesenchymal CSC subpopulations through EMT and *vice versa* through MET, which drives metastasis and tumor relapse (Liu *et al*., 2014). Tumor cells *in vivo* may be able to transiently and reversibly switch between mesenchymal and epithelial states, a process that has been mentioned as epithelial–mesenchymal plasticity (Beerling *et al*., 2016). As such, inhibiting one CSC subpopulation may lead to tumor reconstitution by the other CSC subpopulation. While targeting bulk and both CSC subpopulations is clearly desirable for effective TNBC treatment, mechanistic insights and therapeutic approaches remain elusive (Angeloni *et al*., 2015).

Wnt/β‐catenin signaling has been demonstrated to contribute to breast tumorigenesis and CSC plasticity (Anastas and Moon, 2013; Green *et al*., 2013). β‐Catenin stabilization and nuclear translocation are essential for Wnt signaling. β‐Catenin also acts as an adaptor that links to the cytoplasmic tail of E‐cadherin to mediate cell–cell adhesion (Nelson and Nusse, 2004). The E‐cadherin/β‐catenin complex has been demonstrated to maintain epithelial properties and facilitates self‐renewal of human embryonic stem cells (Chen *et al*., 2010; Huang *et al*., 2015; Li *et al*., 2010; Redmer *et al*., 2011; Tian *et al*., 2011). The intracellular domain of E‐cadherin sequesters β‐catenin to suppress Wnt signaling. Loss of E‐cadherin‐mediated cell–cell contact during epithelial–mesenchymal transition promotes Wnt signaling (Jeanes *et al*., 2008; Serrano‐Gomez *et al*., 2016). However, roles of Wnt signaling in breast cancer remain incompletely understood as it has been shown to either fuel or repress cancer depending on yet to be determined molecular mechanisms (Anastas and Moon, 2013; Green *et al*., 2013).

Over the past few years, Yes‐associated protein (YAP), a downstream effector/transducer of the Hippo pathway, has emerged as a promising anticancer target although it also exhibits a tumor suppressor function in certain diseases (Moroishi *et al*., 2015). YAP has recently been shown to incorporate into the β‐catenin destruction complex to orchestrate Wnt signaling (Azzolin *et al*., 2014). YAP drives cell cycle entry in an E‐cadherin‐ and β‐catenin‐dependent manner (Benham‐Pyle *et al*., 2015) and functions as a mediator of organogenesis and tumorigenesis by stimulating cell proliferation (Yu *et al*., 2015; Zhang *et al*., 2015). Importantly, YAP is also regulated by E‐cadherin (Benham‐Pyle *et al*., 2015). In TNBC cells, E‐cadherin homophilic binding at cell surface impedes the nuclear localization of YAP that is important for the biological activities of YAP (Kim *et al*., 2011). Additionally, α‐catenin, a common binding partner of E‐cadherin which strengthens cellular adhesion, has been demonstrated to bind and sequester YAP in the cytoplasm (Schlegelmilch *et al*., 2011). However, it is unknown whether epithelial–mesenchymal plasticity in cancer affects Wnt and YAP signaling and CSC phenotypes. A strategy for therapeutic blockage of Wnt and YAP to treat TNBC in both epithelial and mesenchymal states remains largely unexploited.

In this study, we demonstrated that YAP signaling was upregulated in mesenchymal‐like TNBC with enriched CD44^high^/CD24^−/low^ CSC subpopulation while Wnt/β‐catenin was upregulated in epithelial‐like TNBC with enriched ALDH^+^ CSC subpopulation. Importantly, the mesenchymal and epithelial TNBC exhibited disparate responses to Wnt and YAP inhibitions and only dual inhibition is capable of effectively suppressing both CD44^high^/CD24^−/low^ and ALDH^+^ CSC populations. These findings were corroborated using patient tumor samples and clinical databases. Furthermore, in a human xenograft model, dual inhibition of Wnt with ICG‐001 and YAP with simvastatin effectively attenuated both mesenchymal and epithelial TNBC tumor burden, diminished both CD44^high^/CD24^−/low^ and ALDH^+^ CSC subpopulations, and reduced tumorigenicity after secondary transplantation. These results suggest that Wnt signaling and YAP signaling are dynamically changed during EMT/MET interconversion and dual inhibition using FDA‐approved drugs can be a viable approach for the treatment of TNBC.

## 2 Materials and methods

### 2.1 Cell culture and reagents

MDA‐MB‐231 breast cancer cells were purchased from the American Type Culture Collection (ATCC, Manassas, VA, USA) and maintained in DMEM‐F12 media supplemented with 10% fetal bovine serum (FBS; HyClone, Logan, UT, USA) and 1% penicillin/streptomycin. SUM149 breast cancer cells were obtained from Asterand (Detroit, MI, USA) and cultured in Hams F‐12 media (Mediatech, Manassas, VA, USA) containing 5% FBS, 5 μg·mL^−1^ insulin, 1 μg·mL^−1^ hydrocortisone, 10 mm HEPES, and 1% penicillin/streptomycin. Cells were cultured at 37 °C in a 5% CO_2_ incubator. ICG‐001 was purchased from CalBiotech (El Cajon, CA, USA) and simvastatin from Caymen Chemicals (Ann Arbor, MI, USA). Insulin, hydrocortisone, HEPES, and bovine serum albumin were purchased from Sigma‐Aldrich (St. Louis, MO, USA).

### 2.2 Tet‐ON inducible gene expression of E‐cadherin

MDA‐MB‐231 E‐cadherin^high^ cells (epithelial‐like, Epi) were generated using a lentiviral vector (pLVX‐Tight‐Puro, Clontech, Mountain View, CA, USA) containing an E‐cadherin gene insert, and control MDA‐MB‐231 E‐cadherin^−/low^ cells (mesenchymal‐like, Mes) were generated using an empty lentiviral vector of pLVX‐Tight‐Puro. Stable clones were selected after 3 days using G418 (Clontech) and puromycin dihydrochloride (Thermo Fisher, Waltham, MA, USA) at a concentration of 1000 and 1 μg·mL^−1^, respectively, for 14 days. For maintenance, 250 μg·mL^−1^ of G418 and 0.25 μg·mL^−1^ of puromycin were added in the culture medium. E‐cadherin expression was activated by adding 1 μg·mL^−1^ doxycycline hydrochloride (Thermo Fisher) to the cell culture every 2–3 days. E‐cadherin levels were examined following RNA extraction by RT‐qPCR and protein levels by western blotting.

### 2.3 Primary normal mammary and breast cancer tissue fragments

Surgical tissues from three patients with TNBC undergoing routine surgical procedures were obtained and used in the experiments. The protocol was approved by the Ottawa Hospital Research Ethics Board (Protocol# 20120559‐01H). Normal mammary tissues or areas containing tumor were identified by gross pathologic examinations. Approximately 2 mm cores were obtained using a sterile biopsy punch that was further sliced with a scalpel to obtain approximately 2 × 1 mm tumor slices (Dayekh *et al*., 2014; Sulaiman *et al*., 2016). The slices were randomized, and three slices were placed into each well of 24‐well plate and cultured in DMEM‐F12 medium supplemented with 10% FBS, 1% penicillin/streptomycin, 1 μg·mL^−1^ insulin, 0.5 ng·mL^−1^ hydrocortisol, and 3 ng·mL^−1^ epidermal growth factor. These primary tissue fragments were treated with the same concentrations of inhibitors as used in the breast cancer cell lines, followed by a viability assay and flow cytometric analysis. The patient‐derived xenograft sample HCI‐001 was obtained from University of Utah and cultured in the same conditions as clinical samples.

### 2.4 Flow cytometry analysis

Dissociated cancer cells were filtered through a 4‐μm strainer and suspended in PBS supplemented with 2% FBS and 2 mm EDTA. One microlitre of mouse IgG (1 mg·mL^−1^) was added and incubated at 4 °C for 10 min. Afterward, the cells were resuspended in 1× binding buffer (eBioscience, San Diego, CA, USA) and incubated with Annexin V (eBioscience) for 15 min at room temperature. Antibodies were added according to the manufacturer's instructions. Apoptosis was determined using Annexin V‐PE‐Cy7 Apoptosis Detection Kit (eBioscience). ALDH activity was determined using ALDEFLUOR (StemCell Technologies, Vancouver, BC, Canada) with a DEAB control. Anti‐CD44 (APC) and anti‐CD24 (PE) (BD Pharmingen) antibodies were used. Lastly, the cells were washed twice with additional ALDEFLUOR assay buffer and 7‐aminoactinomycin D (7‐AAD; eBioscience) was added to exclude dead cells. Flow cytometry was performed on a Cyan‐ADP 9 and the BD LSRFortessa. Data were analyzed with flowjo software (Ashland, OR, USA).

### 2.5 Soft agar colony formation

In a 12‐well plate, the base layer consisted of 0.6% agarose gel containing DMEM/F12 media. The cell layer consisted of 0.35% agarose gel containing DMEM/F12 media and 5 × 10^3^ MDA‐ MB‐231 cells. Plates were incubated at 37 °C in 5% CO_2_ for 21 days. Cell viability was then determined through 3‐(4,5‐dimethylthiazol‐2‐yl)‐2,5‐diphenyl tetrazolium bromide (MTT, 1 mg·mL^−1^) staining. Colonies were then counted (> 100 μm in diameter). All experiments were performed in triplicate, and data are presented as means ± SD.

### 2.6 Western blot analysis

Cells were harvested, washed with PBS, and lysed with lysis buffer supplemented with protease inhibitors (Roche, Sainte‐Agathe‐Nord, QC, Canada). After the protein concentrations were determined using a Bio‐Rad DC protein assay kit (Bio‐Rad, Hercules, CA, USA), samples were then normalized and denatured. The samples were then loaded into an 8% polyacrylamide gel and separated by SDS/PAGE followed by transference to a PVDF membrane. Proteins were identified by incubation with primary antibodies followed by horseradish peroxidase‐conjugated secondary antibodies and an enhanced chemiluminescence solution (Thermo Scientific, Waltham, MA, USA). Antibodies used in this study include the following: anti‐YAP1(1 : 1000, Cat: 4912; Cell Signaling, Cambridge, MA, USA), anti‐CD44 (8E2) monoclonal antibody (1 : 1000, Cat: 5640; Cell Signaling), anti‐ALDH1A1 (1 : 1000, Cat: ab105920; Abcam, Toronto, ON, Canada), anti‐Klf4 (1 : 1000, Cat: ab72543; Abcam), anti‐β‐catenin (1 : 1000, Cat: 610153, Clone 14; BD, Mississauga, ON, Canada), anti‐active β‐catenin (1 : 500, Cat: 05665, Clone 8E7; Millipore, Billerica, MA, USA), and anti‐α‐tubulin monoclonal antibody (1 : 500, Cat: T9026; Sigma‐Aldrich).

### 2.7 Quantitative real‐time PCR

Total RNA was extracted using RNeasy kit (Qiagen, Toronto, ON, Canada) and real‐time qPCR (RT‐qPCR) analysis was performed using Bio‐Rad MyiQ (Bio‐Rad) as previously described (Jia *et al*., 2017; Sulaiman *et al*., 2016). The conditions for RT‐qPCR reactions were one cycle at 95 °C for 20 s followed by 45 cycles at 95 °C for 3‐s and annealing at 60 °C for 30 s. Results were normalized to the housekeeping gene 18S ribosomal RNA (18S) or GAPDH. Relative expression level of genes from different groups was calculated with the ^2ΔΔ^CT method and compared with the expression level of appropriate control cells. Specific primer sequences for individual genes are listed in Table S1.

### 2.8 siRNA knockdown

siRNA for E‐cadherin (#4392420), β‐catenin, and the Silencer Select Negative Control #1 siRNA (Scramble, #4390843) were purchased from Thermo Scientific as SMARTpools. YAP1 silencer^®^ select siRNA was also purchased from Thermo Scientific (ID: s20368). For siRNA transfections, cells were transfected with these oligos using Lipofectamine RNAiMAX reagent (Invitrogen, Carlsbad, CA, USA) according to the manufacturer's instructions. After transfection, efficiency was determined through western blot or RT‐qPCR.

### 2.9 Lentiviral transduction of short hairpin RNA, generation of transgenic Wnt reporter 7xTCF‐eGFP cell lines, and β‐catenin/TCF‐eGFP reporter assays

pLKO.1 puro shRNA β‐catenin was a gift from Bob Weinberg (Addgene plasmid # 18803), shYAP1 was a gift from William Hahn (Addgene plasmid # 42540), and scrambled shRNA was a gift from David Sabatini (Addgene plasmid 1864) (Onder *et al*., 2008; Rosenbluh *et al*., 2012; Sarbassov *et al*., 2005). β‐Catenin/TCF/LEF‐dependent reporter plasmid (7xTcf‐eGFP//SV40‐PuroR, 7TGP) containing seven Tcf/Lef‐binding sites and a puromycin resistance gene was a gift from Nusse (Addgene plasmid 24305). Lentiviral production was carried out as previously described (Jia *et al*., 2016; Sulaiman *et al*., 2016). 10‐cm dishes were seeded with 6 × 10^6^ 293T cells overnight. Afterward, 8 μg of lentivirus vector, 5.4 μg of the psPax2 envelope plasmid, and 3.6 μg of the packaging plasmid (pMD2.G) were used. The medium was replaced overnight, and after 48 h, the lentiviral supernatant was harvested, filtered through a 0.45 μm PES filter, and concentrated with Lenti‐X concentrator (Clontech) according to the manufacturer's instruction. When SUM 149‐PT cells or Mes‐ or Epi‐MDA‐MB‐231 cells in six‐well plates reached 40–50% confluence, 1 mL of concentrated lentiviral supernatant and 8 μg·mL^−1^ of polybrene were added for 24 h, followed by puromycin selection. The expression levels of TCF‐eGFP were determined by flow cytometry.

### 2.10 Cell viability assays

Cell viability analysis was carried out as previously described (Jia *et al*., 2016; Sulaiman *et al*., 2016). Cells were seeded into 12‐well plates (1.5 × 10^4^ cells/well). After 120 h of treatment, Alamar blue viability analysis was performed by incubation with 10% Alamar blue reagent (Thermo Fisher Scientific) for 4 h. Fluorescence was measured at 560 nm excitation and 590 nm emission. Cell viability was also determined through 3‐(4,5‐dimethylthiazol‐2‐yl)‐2,5‐diphenyl tetrazolium bromide (MTT, 1 mg·mL^−1^) staining after incubation for 4 h. Absorbance was measured at 570 nm.

### 2.11 ICG‐001 and simvastatin concentrations selected for the *in vitro* experiments according to the pharmacological studies reported previously

The inhibitor concentrations used in this study for *in vitro* experiments were selected according to the published pharmacological studies. In a phase I clinical trial, 18 patients were given a continuous infusion of the ICG‐001/PRI‐724 for 7 days with dose escalations from 40 to 1280 mg·m^−2^ per day (El‐Khoueiry *et al*., 2013). One patient developed dose‐limiting toxicity of hyperbilirubinemia. The recommended phase 2 dose for ICG‐001/PRI‐724 was 905 mg·m^−2^ based on the incidence of adverse events at 1280 mg·m^−2^ and the plateau in pharmaceutical kinetic parameters (El‐Khoueiry *et al*., 2013). The median *C*_max_ and AUC 0‐t for C‐82 at 905 mg·m^−2^ per day were 887 ng·mL^−1^ and 262 787 h ng·mL^−1^. Median elimination *T*_"_ was 7.35 h (El‐Khoueiry *et al*., 2013). In another clinical study, up to 160 mg·m^−2^ per day of ICG‐001/PRI‐724 was used for a continuous intravenous infusion over six cycles of 1 week followed by 1 week off. No adverse effects were observed for 40 mg·m^−2^ per day group (with a maximum blood concentration of 692 ± 418 ng·mL^−1^) (Kimura *et al*., 2017). Accordingly, 2.5 μm ICG‐001 (=1372 ng·mL^−1^, molecular weight of ICG‐001/PRI‐724: 568.683) was chosen for *in vitro* experiments in this study, which is close to the recommended maximum blood concentration.

Simvastatin is a FDA‐approved drug that has been widely used for the treatment of hypercholesterolemia with up to 80 mg of an oral dosage per day. When taking 20 mg of simvastatin, patient's blood concentration could achieve 28 ng·mL^−1^ with a half‐life of 5.5 h (Tao *et al*., 2016). Oral intake of 40 mg simvastatin was used in another study, resulting in a maximum blood concentration of 34 ng·mL^−1^ (Bellosta *et al*., 2004). Accordingly, 100 nm (=41.86 ng·mL^−1^) of simvastatin (molecular weight = 418.566) was chosen for our *in vitro* experiments.

### 2.12 Xenograft tumor growth

Athymic nude mice were obtained from Charles River Laboratories (Senneville, QC, Canada). The MDA‐MB‐231 breast cancer cells were mixed 1 : 1 with Matrigel and injected under aseptic conditions into the mammary fat pads (*n *=* *4 for each group, 2 × 10^6^ cells per fat pad). When the tumor reached a mean diameter of ~ 3 mm, mice were intraperitoneally injected daily with the vehicle, ICG‐001 (100 mg·kg^−1^ per day), simvastatin (5 mg·kg^−1^ per day), or both for 15 days. At the end of drug treatment, mice were humanely euthanized and tumors were harvested for further analyses and secondary transplantation.

### 2.13 Secondary transplantation of nude mouse model

Tumors were minced using a scalpel and incubated in DMEM media containing collagenase/hyaluronidase (StemCell Technologies, #07912) at 37 °C for 60 min. Afterward, the solution was passed through a 40‐μm nylon mesh for the creation of a single cell solution. The treated tumors were inoculated into one of the mammary fat pads at a concentration of 10^5^, 10^4^, 10^3^, or 10^2^ cells from the original tumors. Tumor growth and size were measured after 6 weeks of growth.

### 2.14 Clinical database analysis and statistical analysis

Breast cancer datasets from the Cancer Genome Atlas (TCGA, http://cancergenome.nih.gov/), Nature Communications 2016 (Pereira *et al*., 2016), Nature 2012 (Network, 2012), and METABRIC (http://molonc.bccrc.ca/aparicio-lab/research/metabric/) were used and analyzed with cBioportal (http://www.cbioportal.org/index.do). *CTNNB1* and *YAP1* gene repression was defined as mRNA expression levels less than three standard deviations below the mean, and protein repression was defined as being below the mean. Expression data and Kaplan–Meier survival curves were generated using the datasets compiled by May 2017 from the following database IDs: *CTNNB1* and *YAP1* gene repression (2509 patients): http://bit.ly/2hTTYOW, CTNNB1 and YAP1 protein repression (887 patients): http://bit.ly/2jNmIgE. CTNNB1, YAP1, and CDH1 protein analysis (410 patients): http://bit.ly/2pHz5xx. Additionally, the Gene Expression Omnibus2R database was used to analyze a dataset (Dataset: GSE45827) to compare the MDA‐MB‐231 cell line to 41 TNBC patient samples https://www.ncbi.nlm.nih.gov/geo/geo2r/?acc=GSE45827. For all clinical database data, the log‐rank test was performed to determine whether observed differences between groups were statistically significant. Data are expressed as means ± standard deviation (SD) or standard error (SE). Statistical significance was determined using anova or Student's *t*‐test. Results were considered significant when *P <* *0.05, **P <* *0.01, or ***P <* *0.001.

## 3 Results

### 3.1 Epithelial TNBC cells exhibit reduced YAP but increased Wnt/β‐catenin signaling

**Figure 1:**
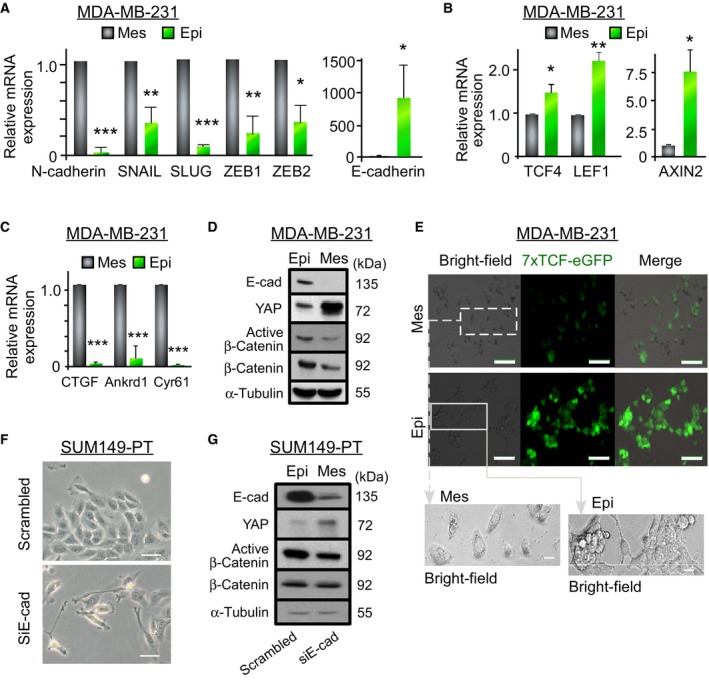
Epithelial‐like, not mesenchymal‐like TNBC cells exhibit upregulated Wnt and downregulated YAP signaling. (A) RT‐qPCR analysis of mesenchymal‐associated genes *N‐CADHERIN*,*SNAIL*,*SLUG*,*ZEB1*,* and ZEB2* as well as *E‐CADHERIN* (*CDH1*) in epithelial‐like MDA‐MB‐231 cells (Epi, generated by overexpression of E‐cadherin) and in mesenchymal‐like control MDA‐MB‐231 cells (Mes). (B) RT‐qPCR analysis of Wnt target genes *TCF4*,*LEF1*,* and AXIN2* in mesenchymal‐like (Mes) and epithelial‐like (Epi) MDA‐MB‐231 cells. (C) RT‐qPCR analysis of YAP target genes *CTGF*,*ANKRD1*,* and CYR61* in mesenchymal‐like (Mes) and epithelial‐like (Epi) MDA‐MB‐231 cells. (D) Representative western blot of E‐cadherin, YAP1, and β‐catenin expression in mesenchymal‐like (Mes) and epithelial‐like (Epi) MDA‐MB‐231 cells. (E) Bright‐field and fluorescence images of mesenchymal‐like (Mes) and epithelial‐like (Epi) MDA‐MB‐231 cells after transfection of the 7xTCF‐eGFP reporter, scale bar = 100 μm. White squares on bright‐field images are enlarged in the bottom panels. The brightness and contrast are adjusted for seeing the shape of the cells, scale bar = 20 μm. (F) Representative phase contrast images of epithelial TNBC SUM 149‐PT cells 48 h after siRNA knockdown of E‐cadherin, scale bar = 50 μm. (G) Representative western blot depicting E‐cadherin, YAP1 and β‐catenin (total β‐catenin and nonphosphorylated at Ser33/37/Thr41 for active β‐catenin) expression in epithelial‐like (Epi) and mesenchymal‐like (Mes) SUM 149‐PT cells 48 h after siRNA E‐cadherin knockdown (siE‐cad). Data represent means ± SE,* n* = 3 for all figures; *P <* *0.05, **P <* *0.01, ***P < 0.001.

E‐cadherin has been used routinely to demarcate epithelial or mesenchymal states (Beerling *et al*., 2016; Liu *et al*., 2014; Tsuji *et al*., 2008). Re‐expression of E‐cadherin in E‐cadherin‐negative mesenchymal‐like MDA‐MB‐231 TNBC cells resulted in an epithelial‐like phenotype (Fig. 1A and Fig. S1: downregulation of a set of mesenchymal genes *N‐CADHERIN*,* SNAIL*,* SLUG*,* ZEB1*, and *ZEB2* and upregulation of a set of epithelial genes *E‐CADHERIN*,* KERATIN 13*,* KERATIN 15*, and *DSP*). Notably, Wnt target genes (*TCF4*,* LEF1*, and *AXIN2*, Fig. 1B) were upregulated in E‐cad+ MDA‐MB‐231 cells while YAP target genes (*CTFG*,* ANKRD1*, and *CYR61*, Fig. 1C) were downregulated. This was corroborated by increased active β‐catenin and diminished YAP1 protein expression (Fig. 1D). Increased Wnt activity in epithelial‐like TNBC cells was also confirmed using a 7×TCF‐eGFP Wnt reporter that contains seven TCF/LEF consensus binding sites upstream of a promoter expressing GFP (Fig. 1E) (Fuerer and Nusse, 2010). Consistently, siRNA knockdown of E‐cadherin in epithelial‐like SUM 149 cells (an E‐cadherin^high^ inflammatory TNBC line) led to a mesenchymal‐like morphology (Fig. 1F), an increase in YAP expression, and a decrease in active β‐catenin protein corroborated by the diminished 7xTCF‐eGFP Wnt reporter activity (Fig. 1G and Fig. S2). Thus, an epithelial phenotype inhibits YAP while promoting Wnt signaling in TNBC.

### 3.2 Epithelial and mesenchymal TNBC cells associate with distinct CSC properties

**Figure 2:**
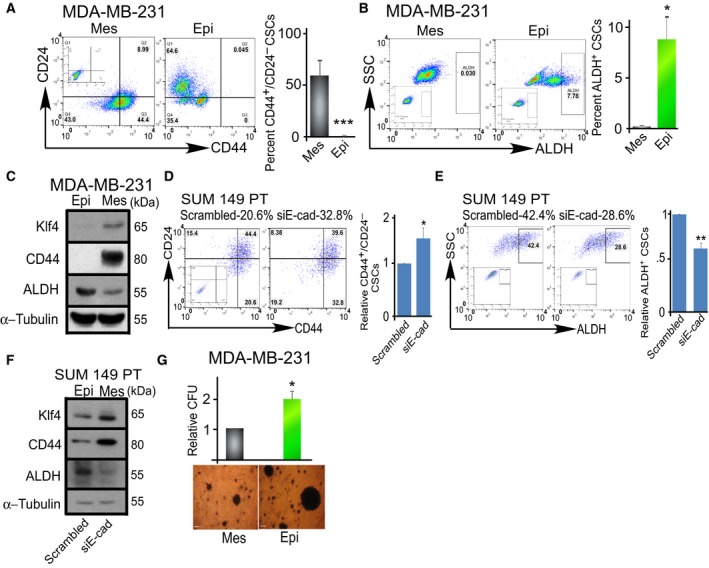
Epithelial‐like and mesenchymal‐like TNBC cells display distinct CSC properties. (A, B) Flow cytometric analysis of CD44^high^/CD24^low^ and ALDH ^+^ CSC subpopulations in mesenchymal‐like (Mes) and epithelial‐like (Epi) MDA‐MB‐231 cells (generated by overexpression of E‐cadherin in mesenchymal‐like MDA‐MB‐231 cells). Insets within flow cytometric plots depict isotype controls for CD44/CD24 (A), or DEAB control (B) for ALDH. (C) Representative western blot depicting pluripotency‐related proteins: Klf4, CD44, and ALDH in mesenchymal‐like (Mes) and epithelial‐like (Epi) MDA‐MB‐231 cells. (D, E) Flow cytometric analysis of CD44^high^/CD24^low^ and ALDH ^+^ CSC subpopulations in the epithelial‐like SUM 149‐PT cells (Epi, scrambled) and mesenchymal‐like (Mes, 48 h after knockdown with siE‐cadherin, siE‐cad). Insets within flow cytometric plots depict isotype controls for CD44/CD24 (A), or DEAB control (B) for ALDH. (F) Representative western blot depicting pluripotency‐related proteins: Klf4, CD44, and ALDH in epithelial‐like SUM 149‐PT cells (Epi, scrambled, transfected with scrambled oligos) and mesenchymal‐like (Mes, 48 h after transfection with siRNA E‐cadherin, siE‐cad). (G) Soft agar colony formation assay to evaluate colony‐forming potential of mesenchymal‐like (Mes) and epithelial‐like (Epi, overexpression of E‐cadherin) MDA‐MB‐231 cells (5000 cells/well). Cells were seeded in soft agar and cultured for 21 days, and colonies were counted after staining with MTT for viability. Scale bar = 100 μm. Data represent means ± SD,* n* = 3 for all figures; *P <* *0.05, **P <* *0.01, ***P < 0.001.

The existence of interconvertible mesenchymal and epithelial populations and CSCs in breast cancer has been associated with drug resistance, metastasis, and diminished survival (Charafe‐Jauffret *et al*., 2010; Li *et al*., 2008; Liu *et al*., 2014; Yan *et al*., 2016). We therefore asked whether conversion between mesenchymal and epithelial phenotypes in TNBC also displayed different CSC phenotypes. Indeed, mesenchymal MDA‐MB‐231 cells contained substantial CD44^high^/CD24^−/low^ but almost undetectable ALDH^+^ CSCs. After conversion to an epithelial phenotype, E‐cad+ MDA‐MB‐231 cells possessed abundant ALDH^+^ CSCs with diminished CD44^high^/CD24^−/low^ CSCs (Fig. 2A,B, flow cytometry). Consistently, western blot showed increased ALDH and diminished CD44 and pluripotency marker Klf4 after MET (Fig. 2C). High expression of Klf4 in breast mesenchymal cells has been associated with metastasis, CSC self‐renewal, and tumorigenicity (Okuda *et al*., 2013; Yu *et al*., 2011). A similar trend was also seen after partial knockdown of E‐cadherin in epithelial SUM149 TNBC cell line (Fig. 2D–F). Epithelial CSCs have been associated with enhanced proliferative properties (Liu *et al*., 2014). Indeed, more colonies were observed in epithelial TNBC cells in comparison with mesenchymal counterparts as determined by an *in vitro* colony‐forming assay (Fig. 2G). It seems that epithelial and mesenchymal TNBC cells associate with distinct CSC properties.

### 3.3 Dual knockdown of Wnt and YAP inhibits mesenchymal and epithelial bulk and CSC subpopulations

**Figure 3:**
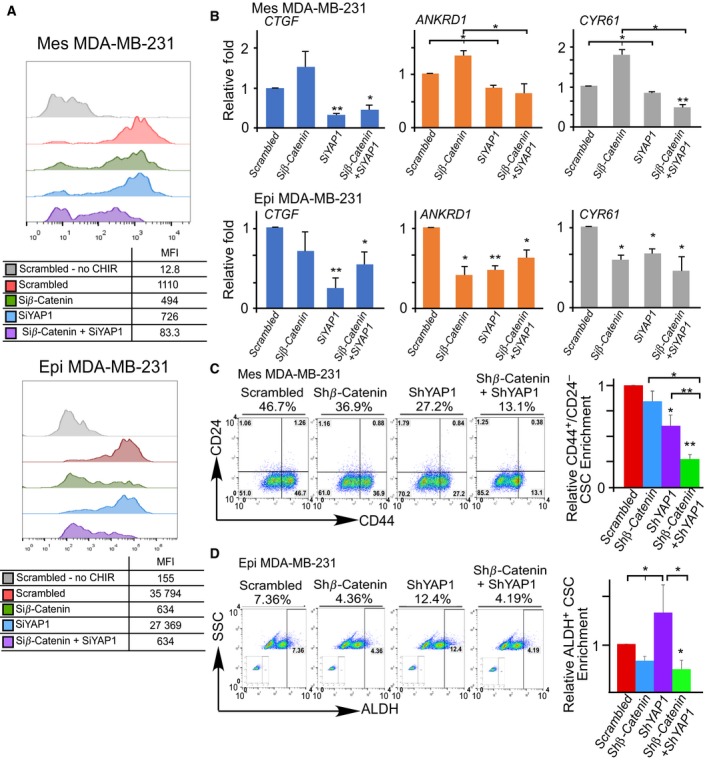
Dual knockdown of Wnt and YAP inhibits mesenchymal and epithelial bulk and CSC subpopulations. (A) Representative flow cytometric analysis of 7xTCF‐eGFP Wnt reporter activity (MFI: median fluorescent intensity) in mesenchymal‐like (Mes) and epithelial‐like (Epi, overexpression of E‐cadherin) MDA‐MB‐231 cells 48 h after siRNA knockdown of β‐catenin and/or YAP1. Cells were exposed to 3 μm CHIR99021 (CHIR, a GSK3 inhibitor activating Wnt signaling) and compared to scrambled + DMSO vehicle and scrambled + CHIR99021 controls. (B) RT‐qPCR analysis of YAP target genes: *CTGF*,*ANKRD1*, and *CYR61* after β‐catenin and/or YAP1 siRNA knockdown in mesenchymal‐like (Mes) or epithetical‐like (Epi) MDA‐MB‐231 cells. Data represent means ± SE. (C,D) Flow cytometric analysis of the CD44^high^/CD24^low^ and ALDH ^+^ CSC subpopulations in the mesenchymal‐like (Mes) and epithelial‐like (Epi) MDA‐MB‐231 cells after shRNA knockdown of β‐catenin and/or YAP1. Insets within flow cytometric plots depict DEAB control for ALDH baseline determination. Data represent means ± SD;* n* = 3 for all figures; *P <* *0.05, **P <* *0.01, ***P < 0.001, in comparison with the indicated or scrambled groups.

We then investigated whether dual knockdown of Wnt and YAP leads to inhibition of both epithelial and mesenchymal bulk and CSC subpopulations. In epithelial TNBC, Wnt reporter assays showed that β‐catenin knockdown (i.e., Wnt inhibition), but not YAP knockdown, effectively repressed Wnt signaling, equivalent to dual knockdown (Fig. 3A). In mesenchymal TNBC cells, however, knockdown of either β‐catenin or YAP only moderately suppressed Wnt signaling, whereas dual knockdown exhibited higher efficacy (Fig. 3A). Interestingly, β‐catenin knockdown (Fig. S3 showing knockdown efficiency) inhibited the expression of YAP target genes in epithelial TNBC cells but upregulated the expression of YAP target genes in their mesenchymal counterparts (Fig. 3B). Unexpectedly, while siRNA knockdown of YAP1 effectively inhibited CD44^high^/CD24^−/low^ CSC subpopulation in mesenchymal TNBC, it increased ALDH^+^ CSCs in epithelial TNBC cells. In contrast, siRNA knockdown of β‐catenin was more effective in inhibiting ALDH^+^ CSCs in an epithelial state but less effective in suppressing CD44^high^/CD24^−/low^ CSCs in a mesenchymal state. These data suggest that Wnt and YAP inhibitions alone exhibit differential effects on mesenchymal and epithelial CSCs. As a result, dual knockdown of Wnt and YAP was a more effective approach to inhibit both CD44^high^/CD24^−/low^ and ALDH^+^ CSC subpopulations in both mesenchymal and epithelial states (Fig. 3C,D).

### 3.4 Combination of ICG‐001 and simvastatin treatment inhibits epithelial and mesenchymal TNBC bulk and CSC populations *in vitro*

**Figure 4:**
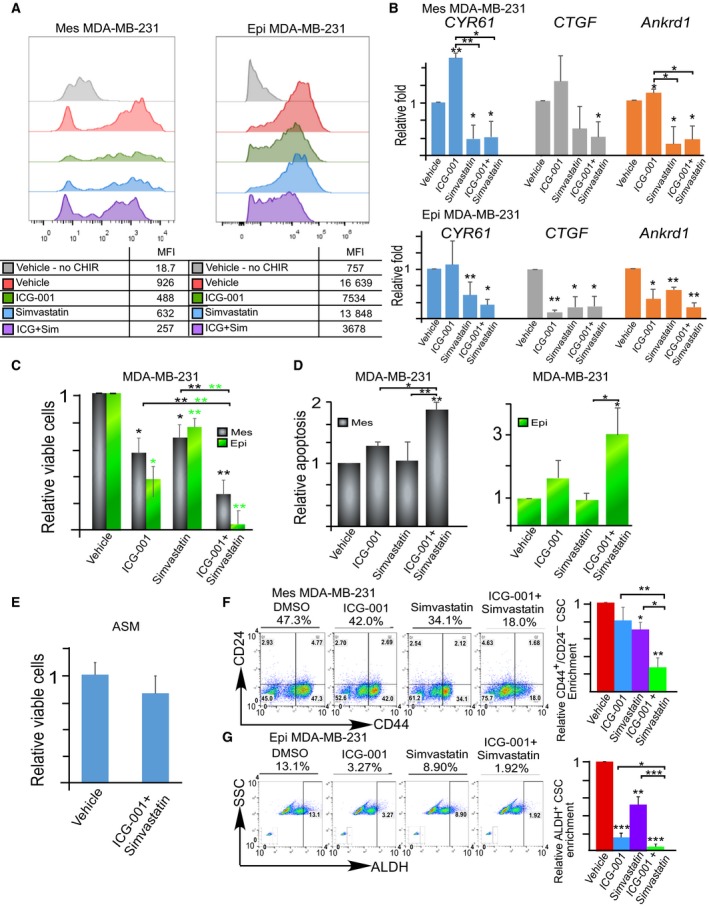
Dual inhibition of YAP and Wnt signaling with small molecules suppresses both mesenchymal‐ and epithelial‐like bulk and CSC populations. (A) Representative flow cytometric analysis of 7xTCF‐eGFP Wnt reporter activity in mesenchymal‐like (Mes) and epithelial‐like (Epi, overexpression of E‐cadherin) MDA‐MB‐231 cells after 48 h of treatment with the vehicle (DMSO), ICG‐001 (5 μm) and/or simvastatin (100 nm). Cells were exposed to 2–3 μm CHIR99021 (CHIR, a GSK3 inhibitor activating Wnt signaling) and compared to vehicle control +/− CHIR99021. (B) RT‐qPCR analysis of YAP target genes (*CTGF*,*ANKRD1*, and *CYR61*) 48 h after treatment with vehicle (DMSO), ICG‐001 (2.5 μm), and/or simvastatin (100 nm) in mesenchymal‐like (Mes) and epithelial‐like (Epi) MDA‐MB‐231 cells. Data represent means ± SE. (C) MTT viability analysis of mesenchymal‐like (Mes) and epithelial‐like (Epi) MDA‐MB‐231 cells after 120 h of exposure to vehicle (DMSO), ICG‐001 (2.5 μm), and/or simvastatin (100 nm). (D) Flow cytometry analysis of apoptosis (Annexin V^+^/7AAD ^+^) of mesenchymal‐like (Mes) and epithelial‐like (Epi) MDA‐MB‐231 cells after 120 h of exposure to vehicle (DMSO), ICG‐001 (2.5 μm), and/or simvastatin (100 nm). (E) Alamar blue viability assays of normal control mammary tissue (ASM) from patient after 120 h of exposure to vehicle (DMSO) or ICG‐001 (2.5 μm) and/or simvastatin (100 nm). (F–G) Flow cytometric analysis of CD44^high^/CD24^low^ and ALDH ^+^ CSCs after 120 h of exposure to ICG‐001 (2.5 μm) and simvastatin (100 nm) in mesenchymal‐like (Mes) and epithelial‐like (Epi) MDA‐MB‐231 cells. Insets within flow cytometric analysis depict DEAB control for ALDH baseline determination. Data represent means ± SD;* n* = 3 for all figures; *P <* *0.05, **P <* *0.01, ***P < 0.001, in comparison with the indicated groups or vehicle control.

To determine the effect of small molecules on dual inhibition of Wnt and YAP signaling in TNBC cells, we used the FDA‐approved ICG‐001/PRI‐724 (a Wnt inhibitor) and simvastatin (inhibiting YAP signaling revealed in 2014 (Wang *et al*., 2014) in addition to other targets). Like that observed in β‐catenin knockdown experiments, ICG‐001 treatment decreased Wnt activity effectively in epithelial TNBC cells (Fig. 4A) and upregulated YAP target genes in mesenchymal TNBC cells (Fig. 4B). Combination of ICG‐001 and simvastatin treatment was able to suppress both Wnt signaling and YAP signaling, reduce cell viability, and promote apoptosis in both mesenchymal and epithelial TNBC cells (Fig. 4C,D, Fig. S4A, and S5A). Flow cytometric analysis showed that the combination treatment also diminished both mesenchymal CD44^high^/CD24^−/low^ and epithelial ALDH^+^ CSC subpopulations compared to vehicle and single inhibitors (Fig. 4F,G, Figs S4B and S5B–C), highlighting the necessity of dual Wnt and YAP suppression. Additionally, normal mammary cells from patient breast tissue were not significantly affected by the combination treatment (Fig. 4E). Hence, the dual inhibition of Wnt and YAP signaling can be an effective approach to halt the growth of epithelial and mesenchymal TNBC cells *in vitro*.

### 3.5 Clinical TNBC patients’ samples exhibit epithelial‐like phenotypes, and dual inhibition of Wnt and YAP signaling suppresses both bulk and CSC populations

**Figure 5:**
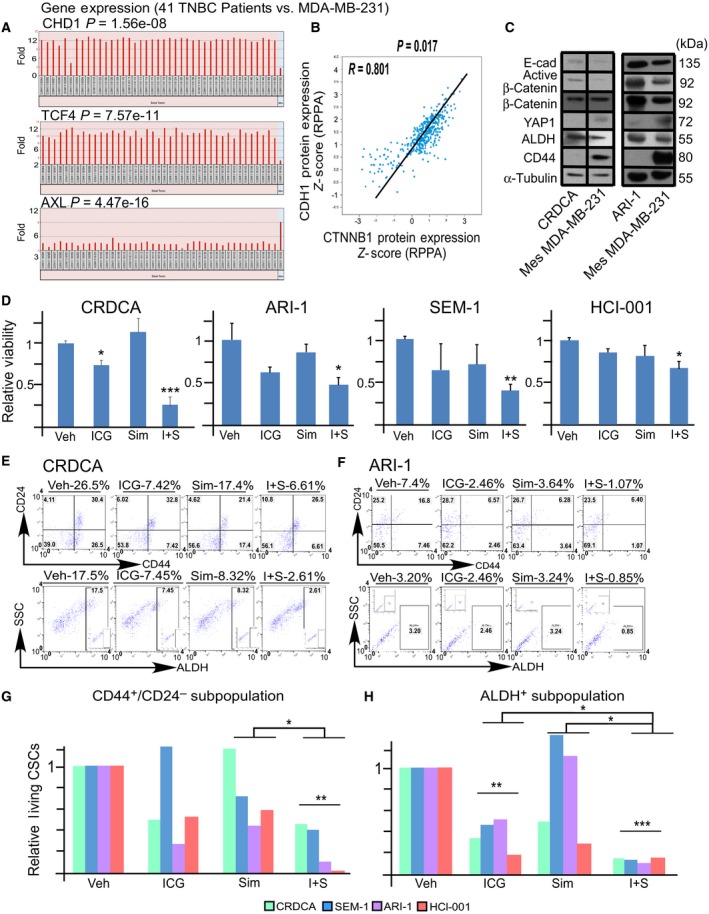
Duel inhibition of YAP and Wnt signaling with small molecules effectively inhibits TNBC patients’ bulk and CSCs. (A) Wnt and YAP target genes (*TCF4* and *AXL*, respectively) and E‐cadherin (*CDH1*) were detected in 41 TNBC patient samples and mesenchymal‐like MDA‐MB‐231 cell line using Affymetrix U133 Plus 2.0 transcriptome analysis Chips (*n *= 41 patients, *P <* *0.05, **P <* *0.01, ***P < 0.001). (B) Positive Pearson correlation between *CDH1* and *CTNNB1* in protein expression (RPPA) in 410 invasive breast cancer patients’ samples (*P <* *0.05). (C) Representative western blot depicting β‐catenin, YAP, E‐cadherin, and pluripotency‐related proteins (ALDH and CD44) of two patient samples (CRDCA and ARI‐1) and mesenchymal‐like MDA‐MB‐231 cell line. See also Fig. S6 for additional patient's sample. (D) Alamar blue viability analysis of three primary patients’ TNBC samples (CRDCA, SEM‐1 and ARI‐1) and one PDX sample (HCI‐001) after 120 h of exposure to vehicle (DMSO), ICG‐001 (2.5 μm) and/or simvastatin (100 nm). (E–H) Representative flow cytometric analysis of CD44^high^/CD24^low^ and ALDH ^+^ CSC subpopulations in patients’ sample CRDCA and ARI‐1 after 120 h of exposure to vehicle (DMSO), ICG‐001 (2.5 μm) and/or simvastatin (100 nm) (E–F). The relative living CD44^high^/CD24^low^ and ALDH ^+^ CSCs in all clinical samples are tabulated (G–H). Insets within flow cytometric plots depict DEAB control for ALDH baseline determination. All data in Fig. 5 represent means ± SD,* n* = 3–4; *P <* *0.05, **P <* *0.01, ***P < 0.001, in comparison with the indicated groups or vehicle control.

In comparison with mesenchymal MDA‐MB‐231 cell line, almost all 41 primary TNBC tumors (Omnibus2R, Dataset: GSE45827, Accessed July 14, 2017; Barrett *et al*., 2013) showed increased expression of E‐cadherin (*CDH1*) and Wnt target gene *TCF4* but decreased YAP target gene *AXL* (Fig. 5A). Likewise, primary TNBC patients’ tumor samples (CRDCA, SEM‐1, and ARI‐1) also showed increased expression of E‐cadherin, active β‐catenin, and ALDH but decreased expression of YAP and CD44 (Fig. 5C, Fig. S6). It seems that patients’ TNBC samples exhibit a more epithelial‐like phenotype in comparison with the mesenchymal‐like MDA‐MB‐231 TNBC cell line. In addition, the E‐cadherin protein levels were positively correlated with β‐catenin expression in 410 breast cancer patients’ tumor samples (Fig. 5B, cBioportal) (Cancer Genome Atlas Network, 2012), consistent with the data obtained from TNBC cell lines in Fig. 1.

We then treated three patients’ tumor fragments and one patient‐derived‐xenograft (PDX) fragment (DeRose *et al*., 2011) with Wnt and/or YAP inhibitors. In all TNBC patients’ samples, dual inhibition of Wnt and YAP reduced cell viability (Fig. 5D), and inhibited both CD44^high^/CD24^−/low^ and ALDH^+^ CSC subpopulations than single inhibition alone (Fig. 5E–H).

### 3.6 Dual inhibition of Wnt and YAP signaling is capable of retarding tumor growth and inhibits CSC subpopulations and tumorigenesis *in vivo*

**Figure 6:**
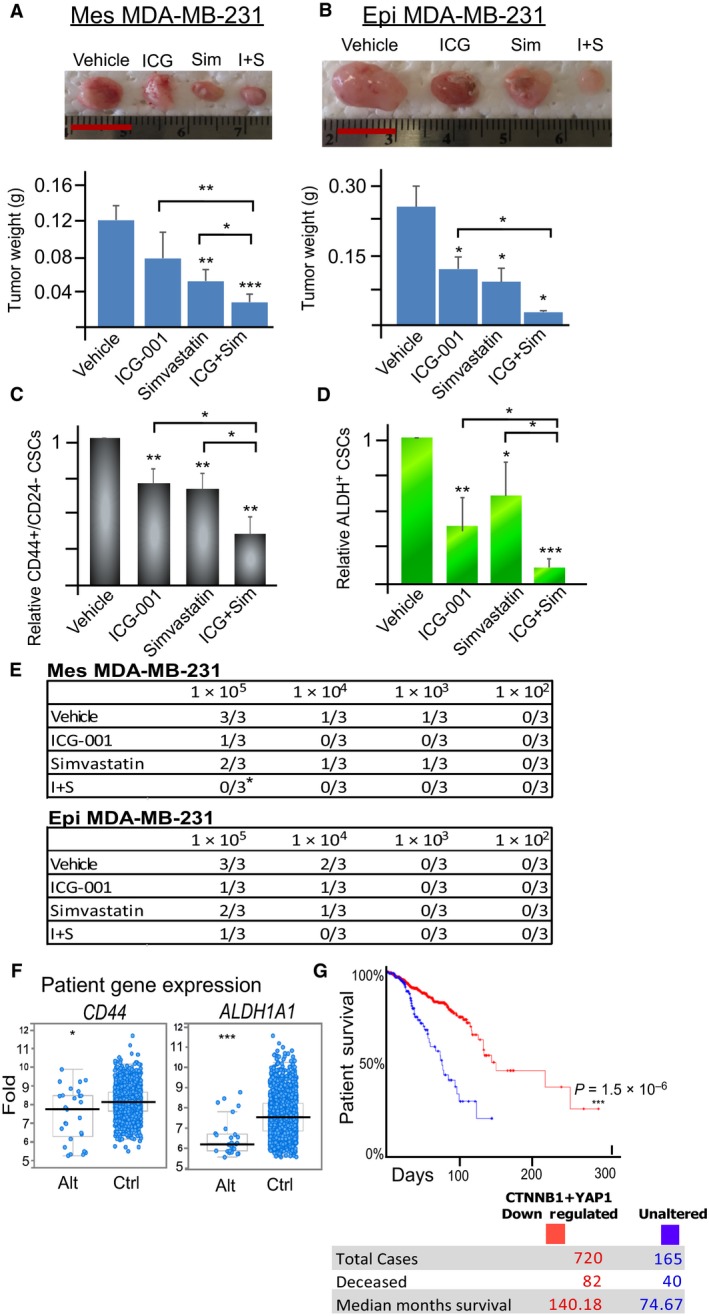
Combination therapy with YAP and Wnt small molecule inhibitors effectively retards tumor growth and reduces CSC enrichment and tumorigenesis *in vivo*; low expression of *CTNNB1* and *YAP1* genes correlates with low expression of CD44^+^ and ALDH1A1^+^ genes in patients’ tumor samples while inversely correlates with improved survival in breast cancer patients. (A,B) Mesenchymal‐like (Mes) and epithelial‐like (Epi) MDA‐MB‐231 TNBC cells were injected into the mammary fat pads of athymic nude mice. When the tumors reached a mean diameter of 3 mm, mice were separated into four groups and intraperitoneally injected daily with the vehicle (DMSO), ICG‐001 (100 mg·g^−1^ per day), simvastatin (5 mg·g^−1^ per day), and ICG‐001 + simvastatin for 15 days. After conclusion of treatments, tumors were harvested from the mice, photographed, and weighed. Data represent means ± SD;* n* = 4 mice for each group; *P <* *0.05, **P <* *0.01. Scale bar = 1 cm. (C, D) Flow cytometric analysis of the living CD44^high^/CD24^low^ and ALDH ^+^ CSC subpopulations of dissociated mesenchymal‐like (Mes) and epithelial‐like (Epi) tumors after 15 days of treatment with vehicle (DMSO), ICG‐001, and/or simvastatin. Insets within flow cytometric plots depict DEAB control for ALDH baseline determination. Data represent means ± SD;* n *= 4 mice for each group; *P <* *0.05, **P <* *0.01, in comparison with the indicated groups or vehicle control. (E) Mesenchymal‐like (Mes) and epithelial‐like (Epi) MDA‐MB‐231 xenografts were dissociated into single cell suspension and retransplanted into the mammary fat pads of new nude mice in serial limiting dilutions (10^5^, 10^4^, 10^3^, or 10^2^ cells per injection). Tumor formation was observed for 6 weeks. (F) Low levels of *CTNNB1* (a pivotal effector of the canonical Wnt signaling pathway) and *YAP1* (YAP signaling) gene expression in breast cancer patients’ samples (Alt) correlates with low levels of *CD44* and *ALDH1A1* gene expression in comparison with those patients’ samples without showing the reduced expression of *CTNNB1* and *YAP1* genes (Ctrl). *N *= 2509 patients with invasive breast cancer, *P <* *0.05, ***P < 0.001. (G) Kaplan–Meier curves for overall survival of the patients with low levels of CTNNB1 (Wnt) and YAP1 (YAP) protein expression in cancer samples (red curve) in comparison with those patients with unaltered expression (blue curve). n = 887, ***P <* *0.001, log‐rank test.

We next determined the effect of combination treatment *in vivo*. Mesenchymal and epithelial MDA‐MB‐231 (overexpressing E‐cadherin) cells were injected into the mammary fat pad of athymic mice. When tumor reached a mean diameter of 3 mm, mice were randomized into four groups and injected intraperitoneally with vehicle, ICG‐001 (100 mg·kg^−1^ per day), simvastatin (5 mg·kg^−1^ per day), or both for 15 days. As expected, the combination treatment reduced tumor burden of both mesenchymal and epithelial TNBC (Fig. 6A,B). To determine CSC pool *in vivo*, we harvested tumors at the end of the treatment and assessed CD44^high^/CD24^−/low^ and ALDH^+^ subpopulation using flow cytometry. As shown in Fig. 6C,D, dual administration of ICG‐001 and simvastatin reduced both CD44^high^/CD24^−/low^ and ALDH^+^ CSC subpopulations in mesenchymal and epithelial‐like TNBC, respectively, in comparison with vehicle or single drug treatments, suggesting the necessity of dual Wnt and YAP inhibition for suppressing CSC subpopulations.

To determine whether co‐administration of ICG‐001 and simvastatin inhibits tumor‐initiating potential, we performed secondary transplantation. We serially diluted tumor cells containing various percentage of CD44^high^/CD24^−/low^ and ALDH^+^ subpopulations isolated from the primary tumors, and transplanted them into athymic nude mice without further treatment for 6 weeks. Tumor cells isolated from mice receiving both ICG‐001 and simvastatin exhibited the least tumor‐initiating capacity in comparison with single treatments and a vehicle control (Fig. 6E). Thus, dual inhibition of Wnt and YAP signaling can reduce tumor burden, but more importantly, it suppresses CSCs and attenuates tumorigenesis in mesenchymal and epithelial TNBC after secondary transplantation.

### 3.7 Low expression of *CTNNB1* and *YAP1* genes correlates with low expression of CD44^+^ and ALDH1A1+ genes and improved survival in breast cancer patients

Analysis of a database containing gene expression of 2509 breast cancer patients using cBioportal (Cerami *et al*., 2012; Gao *et al*., 2013; Pereira *et al*., 2016) showed that in breast tumor samples, decreased gene expressions of *CTNNB1* (a pivotal effector of the canonical Wnt signaling pathway) and *YAP1* (YAP) were accompanied by reduced gene expressions of *CD44* and *ALDH1A1* that are associated with mesenchymal and epithelial CSC phenotypes (Fig. 6F). In addition, analysis of a dataset of 887 patients with invasive breast carcinoma showed that coreduction of CTNNB1 (Wnt) and YAP1 (YAP) protein expression was correlated with improved patients’ survival (Fig. 6G, median survival of 140.18 months *versus* 74.67 months in the unaltered control). Those with either reduced expression of CTNNB1 or YAP1 protein alone showed only a moderate increase in survival (32.66 months by CTNNB1 and 9.53 months by YAP1) in comparison with the unaltered control (Fig. S7).

## 4 Discussion

Epithelial–mesenchymal plasticity and CSCs are key challenges for effective cancer treatment. In this study, we observed that dynamic changes in Wnt and YAP signaling and CSC phenotypes are dependent on epithelial or mesenchymal states. YAP is upregulated in mesenchymal TNBC cells while Wnt upregulated in epithelial TNBC cells. These observations are clearly supported within the TNBC literature. The intracellular domain of E‐cadherin has been shown to mediate YAP nuclear exclusion and β‐catenin activity (Benham‐Pyle *et al*., 2015; Kim *et al*., 2011). Additionally, α‐catenin and 14‐3‐3 proteins are known to associate with YAP and prevent its dephosphorylation via PP2A under the upstream control of E‐cadherin (Schlegelmilch *et al*., 2011).

Importantly, we found that mesenchymal and epithelial TNBC cells exhibited different responses to Wnt and YAP inhibitions. Knockdown of Wnt/β‐catenin upregulated YAP target genes in mesenchymal‐like TNBC cells, which is consistent with a recent report showing that inhibition of Wnt/β‐catenin signaling facilitates YAP/TAZ overexpression‐induced liver growth and tumor initiation (Kim *et al*., 2017). We also found Wnt/β‐catenin knockdown was more effective in suppressing ALDH^+^ CSCs in an epithelial state than in suppressing CD44^high^/CD24^−/low^ CSCs in a mesenchymal state. In contrast, YAP knockdown enriched ALDH^+^ CSCs in epithelial‐like TNBC cells although it potently inhibited CD44^high^/CD24^−/low^ in mesenchymal‐like TNBC cells. These observations suggest that inconsistent results reported in breast cancer cells in response to Wnt or YAP inhibition (Anastas and Moon, 2013; Green *et al*., 2013; Maugeri‐Saccà and De Maria, 2016) may be associated with ineffective CSC targeting due to epithelial and/or mesenchymal states, TNBC EMT/MET plasticity, and YAP and Wnt feedbacks. Dual inhibition of YAP and Wnt signaling on the other hand can suppress both epithelial‐ and mesenchymal‐like bulk and CSC populations without significantly affecting cultured normal mammary tissue fragments *in vitro* and mice *in vivo*, suggesting a favorable approach for this combination therapy. This was supported by the alternations of CD44^high^/CD24^−/low^ and ALDH^+^ CSC subpopulations in both MDA‐MB‐231 and SUM149‐PT cell lines, although the changes in SUM149‐PT cells after siRNA knockdown of E‐cadherin (which induces a mesenchymal‐like phenotype) were not as robust as seen in mesenchymal MDA‐MB‐231 cells. This may be associated with incomplete siRNA silence, recovery of E‐cadherin after knockdown, and/or experimental timing.

We also observed that knockdown of YAP or usage of simvastatin suppressed Wnt signaling. The suppressed Wnt signaling may be associated with the formation of YAP, β‐catenin, and TBX5 complex that is essential for transformation and survival of β‐catenin‐driven cancers (Rosenbluh *et al*., 2012). At present, it is unclear why such an effect is significant in mesenchymal but less in epithelial TNBC cells, warranting further investigations. Nevertheless, as epithelial and mesenchymal cancer cells are interconvertible, simultaneously targeting YAP and Wnt signaling should be taken into consideration in future TNBC treatment.

Administration of ICG‐001 and simvastatin was clinically relevant; both are FDA‐approved drugs for clinical applications with defined pharmacological dynamics/kinetics and have the potential to be readily repurposed in this clinical indication. ICG‐001/PRI‐724 is a fairly specific Wnt inhibitor and is used for the treatment of acute and chronic myeloid leukemia (NCT01606579). Simvastatin is an inhibitor of HMG‐CoA reductase and widely employed as a cholesterol‐lowering drug. In addition, simvastatin has been reported to affect wide plethora of targets including YAP, RhoA, Ras, Akt, mTOR, and JAK2/STAT3 (Fang *et al*., 2013; Wang *et al*., 2016; Wu and Liu, 2008). We have observed that ICG‐001 inhibits TNBC Wnt signaling, and simvastatin suppresses YAP signaling although other off‐target effects coexist. As ICG‐001 and simvastatin exhibited effects resembling YAP1 and β‐catenin knockdown in TNBC cells, it is likely that the biological changes observed in this study are associated with Wnt and YAP inhibitions. This study identifies different expressions of CSC phenotypes and cellular responses to YAP and Wnt targeting associated with mesenchymal or epithelial state. Through dual inhibition of Wnt and YAP signaling, both epithelial and mesenchymal CSC subpopulations can be inhibited and tumorigenesis can be halted after secondary transplantation, which may reduce TNBC recurrence. As simvastatin is commonly prescribed and ICG‐001/PRI‐724 has been approved by FDA for clinical trial evaluation, further investigation of this combination and other Wnt and YAP inhibitors may lead to an effective therapy with reduced toxicity and attenuated CSC enrichment as compared to conventional chemotherapy.

## Author contributions

AS and LW conceived and designed the study. AS, SM, LL, DJ, and SO performed the *in vitro* experiments. AS and SM performed the *in vivo* experiments. AS, SM, and SO analyzed the data. AS drafted the manuscript. LW, CA, JD, SG, and ZY edited the manuscript. AA and CN provided clinical samples for the study. CA, JD, ZY, GJ, HS, YS, SG, and XL provided valuable suggestions and assisted in troubleshooting the experiments. AS, LW, XL, ZY, GJ, HS, CA, YS, and SG conceived or designed the experiments. All authors approved the final version of the manuscript.

## Supporting information


**Fig. S1.** Overexpression of E‐cadherin in mesenchymal‐like MDA‐MB‐231 TNBC cells resulted in an epithelial‐like phenotype.
**Fig. S2.** 7xTCF‐eGFP Wnt reporter activity upon E‐cadherin knockdown in SUM 149‐PT cells.
**Fig. S3.** *CTNNB1* and *YAP1* knockdown efficacy in mesenchymal‐like (Ctrl) and epithelial‐like (E‐cad+) MDA‐MB‐231 TNBC cells.
**Fig. S4.** Suppression of Wnt and pluripotency‐related genes after treatment with ICG‐001 and simvastatin in mesenchymal‐like (Mes) and epithelial‐like (Epi) MDA‐MB‐231 TNBC cells.
**Fig. S5.** Dual inhibition of YAP and Wnt signaling suppresses both mesenchymal and epithelial‐like bulk and CSC populations in epithelial‐like SUM149‐PT TNBC cells.
**Fig. S6.** Western blot analysis of patient TNBC tumor fragment in comparison with MDA‐MB‐231 cell line.
**Fig. S7.** Kaplan–Meier curves for overall survival of the patients with low levels of Wnt (CTNNB1) or YAP (YAP1) protein expression in cancer samples.
**Table S1.** Primers used in RT‐qPCR.
