# Supplementary material for: Dual inhibition of Wnt and Yes‐associated protein signaling retards the growth of triple‐negative breast cancer in both mesenchymal and epithelial states
Source: Mol Oncol. 2018 Feb 21;12(4):423–40. doi: 10.1002/1878-0261.12167 (PMC5891054; doi:10.1002/1878-0261.12167)
Supplement: Supplementary file 1 — Fig. S1. Overexpression of E‐cadherin in mesenchymal‐like MDA‐MB‐231 TNBC cells resulted in an epithelial‐like phenotype. Fig. S2. 7xTCF‐eGFP Wnt reporter activity upon E‐cadherin knockdown in SUM 149‐PT cells. Fig. S3. CTNNB1 and YAP1 knockdown efficacy in mesenchymal‐like (Ctrl) and epithelial‐like (E‐cad+) MDA‐MB‐231 TNBC cells. Fig. S4. Suppression of Wnt and pluripotency‐related genes after treatment with ICG‐001 and simvastatin in mesenchymal‐like (Mes) and epithelial‐like (Epi) MDA‐MB‐231 TNBC cells. Fig. S5. Dual inhibition of YAP and Wnt signaling suppresses both mesenchymal and epithelial‐like bulk and CSC populations in epithelial‐like SUM149‐PT TNBC cells. Fig. S6. Western blot analysis of patient TNBC tumor fragment in comparison with MDA‐MB‐231 cell line. Fig. S7. Kaplan–Meier curves for overall survival of the patients with low levels of Wnt (CTNNB1) or YAP (YAP1) protein expression in cancer samples. Table S1. Primers used in RT‐qPCR. [file MOL2-12-423-s001.pdf]

Supplemental Figure 1

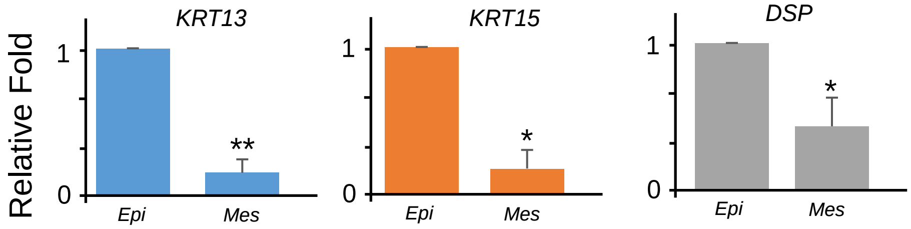

Supplemental Figure 1: Overexpression of E-cadherin in mesenchymal-like MDA-MB-231 TNBC cells resulted in an epithelial-like phenotype

RT-qPCR analysis of epithelial genes *KRT13* (Keratin 13), *KRT15* (Keratin 15) and *DSP* (Desmoplakin) in epithelial-like (Epi) MDA-MB-231 and mesenchymal-like (Mes) cells. Data represent means ± SE, n = 3 for all figures; * $p < 0.05$, ** $p < 0.01$.

Supplemental Figure 2

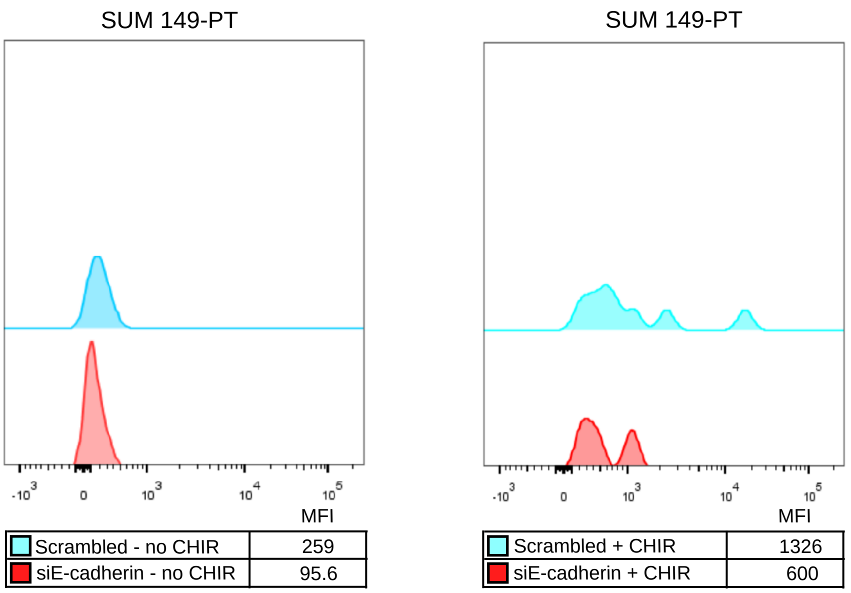

Supplemental Figure 2: 7xTCF-eGFP Wnt reporter activity upon E-cadherin knockdown in SUM 149-PT cells

Representative flow cytometric analysis of 7xTCF-eGFP Wnt reporter activity (MFI: median fluorescent intensity) in SUM 149-PT cells 48 hours after siRNA knockdown of E-cadherin. Cells were exposed to vehicle (left panel) or 3µM CHIR99021 (a GSK3 inhibitor activating Wnt signaling, right panel).

Supplemental Figure 3

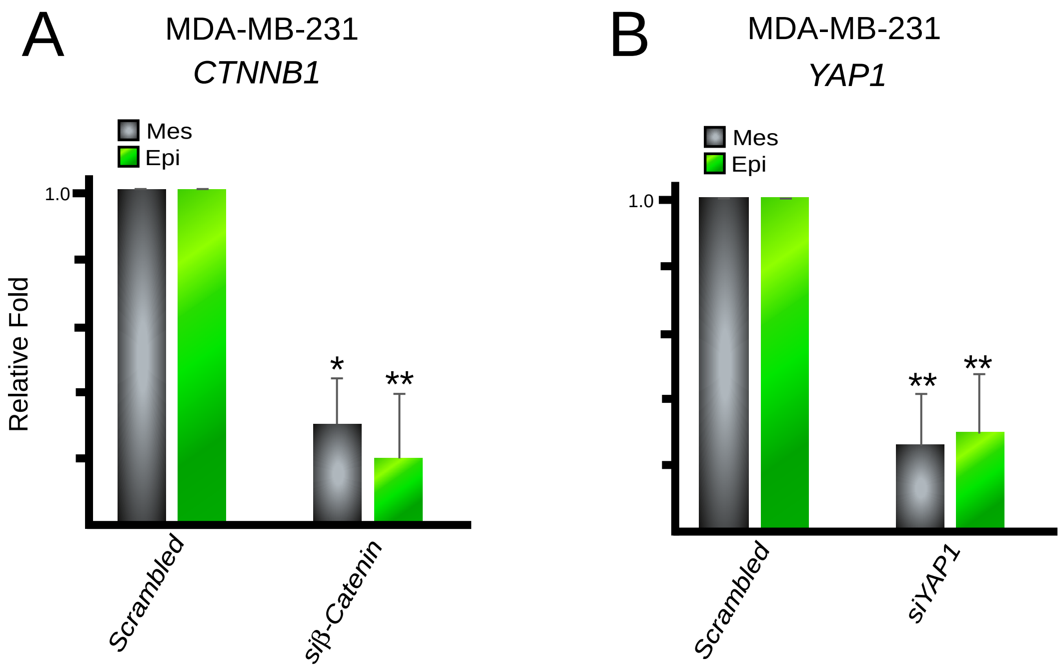

Supplemental Figure 3: *CTNNB1* and *YAP1* knockdown efficacy in mesenchymal-like (Ctrl) and epithelial-like (E-cad+) MDA-MB-231 TNBC cells.

(A-B) RT-qPCR analysis of *CTNNB1* and *YAP1* 48 hours after siRNA knockdown of ß-catenin and YAP1 in mesenchymal-like (Mes) or epithetical-like (Epi) MDA-MB-231 cells. Data represent means ± SE, n = 3 for all figures; * $p < 0.05$, ** $p < 0.01$.

Supplemental Figure 4

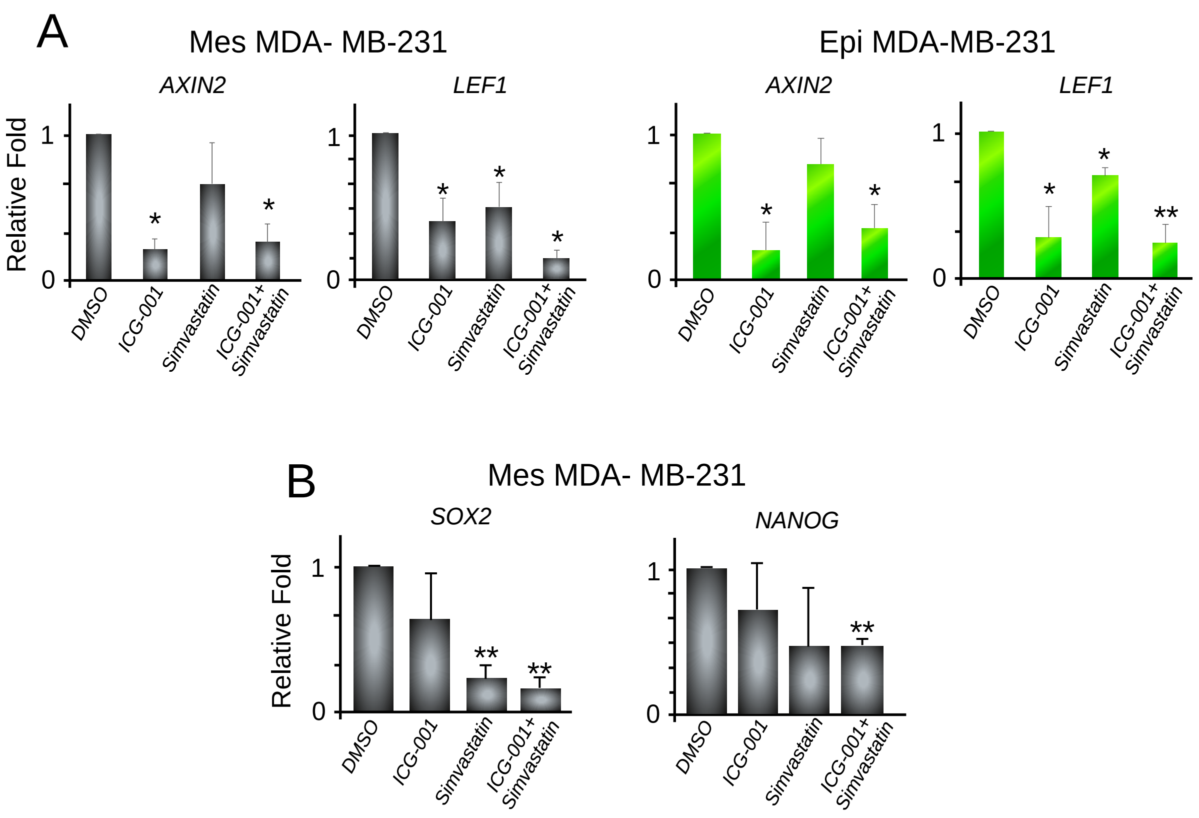

Supplemental Figure 4: Suppression of Wnt and pluripotency-related genes after treatment with ICG-001 and simvastatin in mesenchymal-like (Mes) and epithelial-like (Epi) MDA-MB-231 TNBC cells.

(A) RT-qPCR analysis of Wnt genes *AXIN2* and *LEF1* 48 hours after treatment with vehicle (DMSO), ICG-001 (2.5 μM) and/or simvastatin (100 nM) in mesenchymal-like (Mes) and epithelial-like (Epi) MDA-MB-231 cells.

(B) RT-qPCR analysis of pluripotent genes *SOX2* and *NANOG,* 48 hours after treatment with vehicle (DMSO), ICG-001 (2.5 μM) and/or simvastatin (100 nM) in mesenchymal-like (Mes) and epithelial-like (Epi) MDA-MB-231 cells. Data represent means ± SE, n = 3 for all figures; * $p < 0.05$, ** $p < 0.01$.

Supplemental Figure 5

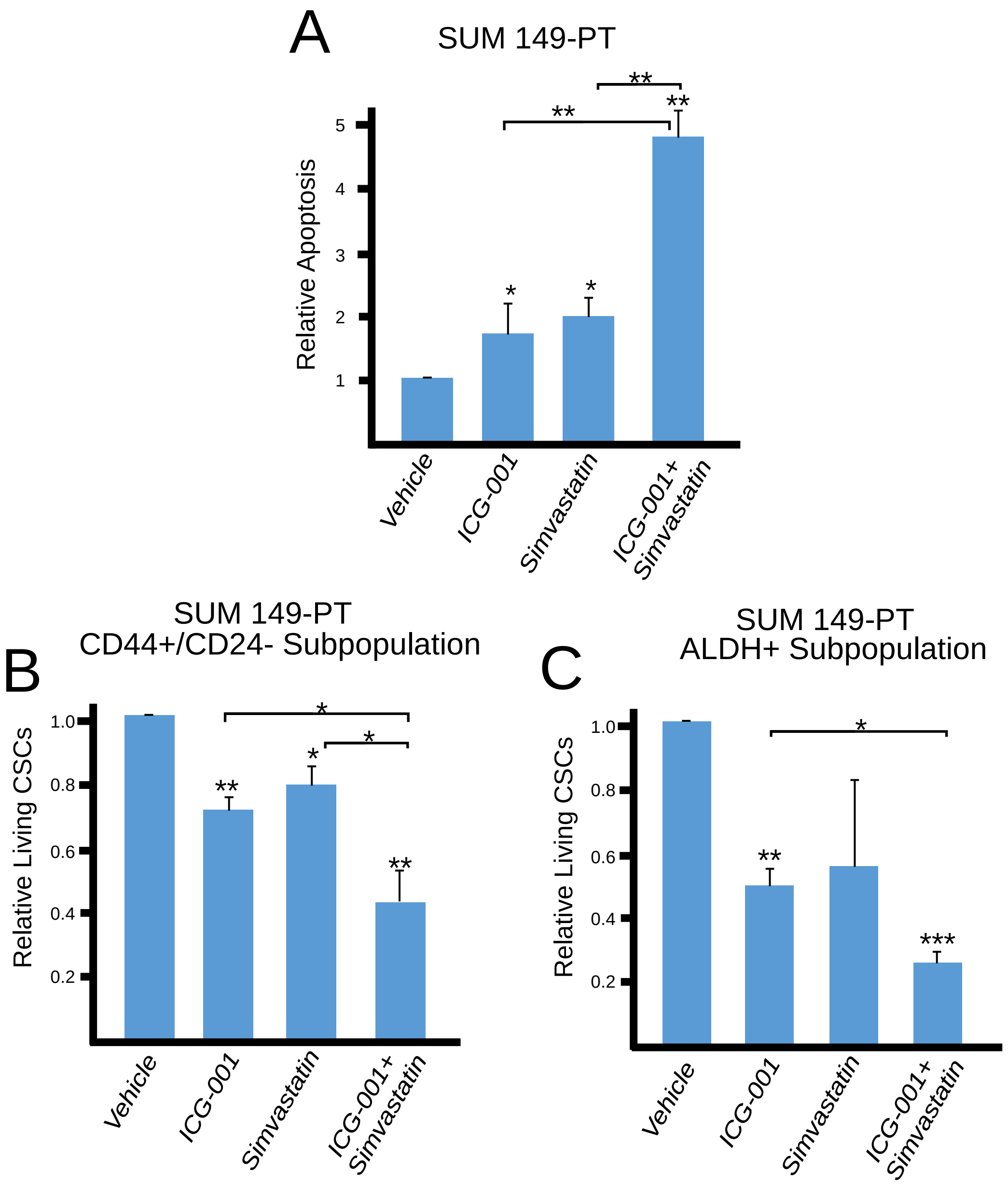

Supplemental Figure 5: Dual inhibition of YAP and Wnt signaling suppresses both mesenchymal and epithelial-like bulk and CSC populations in epithelial-like SUM149-PT TNBC cells.

(A) Flow cytometry analysis of apoptosis (Annexin V+/7AAD+) of SUM149-PT cells after 120 hours of exposure to vehicle (DMSO), ICG-001 (2.5 µM) and/or simvastatin (100 nM).

(B-C) Flow cytometric analysis of $CD44^{high}/CD24^{low}$ and ALDH+ CSCs after 120 hours of exposure to ICG-001 (2.5 µM) and simvastatin (100 nM) in SUM149-PT cells. Data represent means ± SD, n = 3 for all figures; * $p < 0.05$, ** $p < 0.01$, *** $p < 0.001$.

Supplemental Figure 6

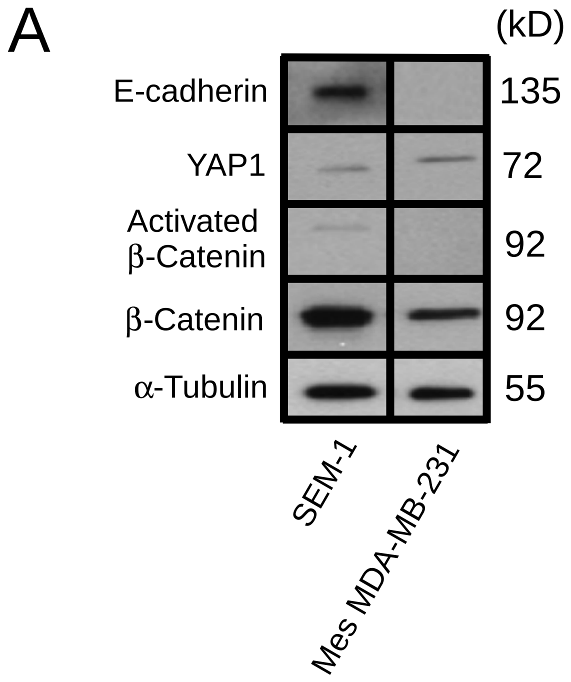

Supplemental Figure 6: Western blot analysis of patient TNBC tumor fragment in comparison to MDA-MB-231 cell line

(A) Representative western blot depicting β-catenin, YAP and E-cadherin expression in patient tumor samples (SEM-1) and the mesenchymal-like MDA MB-231 cell line.

Supplemental Figure 7

A

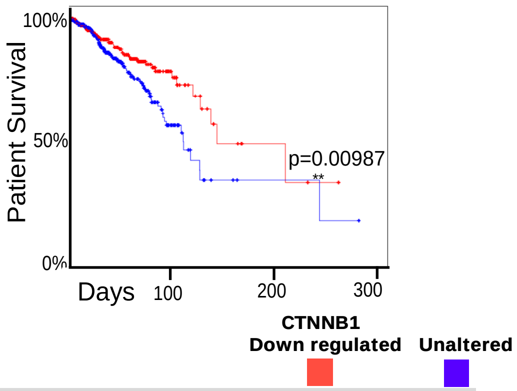

	CTNNB1 Down regulated	Unaltered
Total Cases	389	496
Deceased	45	77
Median Months Survival	146.39	113.73

B

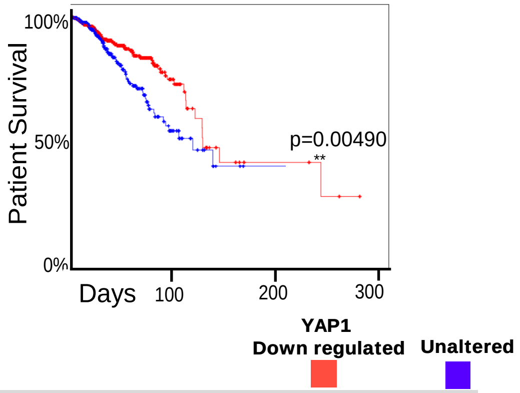

	YAP1 Down regulated	Unaltered
Total Cases	498	387
Deceased	59	63
Median Months Survival	130.06	120.53

Supplemental Figure 7: Kaplan-Meier curves for overall survival of the patients with low levels of Wnt (CTNNB1) or YAP (YAP1) protein expression in cancer samples

(A-B) Kaplan-Meier curves for overall survival of the patients with low levels of CTNNB1 (Wnt) or YAP1 (YAP) protein expression in cancer samples (red curve) in comparison to those patients with unaltered expression (blue curve, n = 885, $^{**}p < 0.01$, log-rank test).

Table S1. Primers used in RT-qPCR

Genes	Forward	Reverse
**18S**	AACCCGTTGAACCCCATT	CCATCCAATCGGTAGTAGCG
**GAPDH**	ACAGTCAGCCGCATCTTCTT	GACAAGCTTCCCGTTCTCAG
**YAP1**	CACCAATTATTTCGGCAGGA	CATCCTGCTCCAGTGTAGGC
**CTGF**	AGGAGTGGGTGTGTGACGA	CCAGGCAGTTGGCTCTAATC
**Cyr61**	AGCCTCGCATCCTATACAACC	TTCTTTCACAAGGCGGCACTC
**Ankrd1**	CACTTCTAGCCCACCCTGTGA	CCACAGGTTCCGTAATGATTT
**TCF4**	GAGGTGGCATGCACTGTC	CGCTGTGTTCATTGGTCTCT
**Axin-2**	TAAGCACCGTCTTGATCGCCCAAT	GTCACGAAACCCATGCGTGTCTCT
**LEF1**	TTCTCCACCCATCCCGAGAAC	CTGAGGCTTCACGTGCATT
**N-cadherin**	GACGGTTCGCCATCCAGAC	TCGATTGGTTTGACCACGG
**E-cadherin**	TGCCCAGAAAATGAAAAAGG	GGATGACACAGCGTGAGAGA
**Snail**	TGCAGGACTCTAATCCAAGTTTACC	GTGGGATGGCTGCCAGC
**Slug**	TGTTGCAGTGAGGGCAAGAA	GACCCTGGTTGCTTCAAGGA
**ZEB1**	GCCAATAAGCAAACGATTCTG	TTTGGCTGGATCACTTTCAAG
**ZEB2**	GGAACACCCCTGGCACAACAAC	GATCGTGGCTTCTGGCCCCATA
**Nanog**	CATGAGTGTGGATCCAGCTTG	CCTGAATAAGCAGATCCATGG
**Sox2**	CATCACCCACAGCAAATGACAGC	TTGCGTGAGTGTGGATGGGATTG
**DSM**	GCTTGCCAACTTCAGAGGTTCT	TTGGAGAATAGCCTGGAGCAGT
**KRT13**	ACACTGCCATGATTCAGACCA	ATAGCGGCACTCCGTCTCT
**KRT15**	AAGACGGAGATCACAGACCTG	TGTCTCGGCCAGTGAGTTCT
